# On the Danger of Curing Certain Diseases

**Published:** 1810-09

**Authors:** 


### On the Danger of curing certain Diseases.

( In Continuation. )

A Young officer, of a hot and sanguine temperament, who, during a long sea voyage had the itch, produced by the gross and salted provisions he was obliged to subsist on, upon his coming on shore rubbed himself with some ointment, whose composition I was ignorant of; the itch disappeared, but he was dangerously ill a long time, vomiting up every thing he took, whether solid or fluid; he was troubled also with a constant hiccough and cardialgia; these symptoms were attended by sleepless nights, want of appetite, slow fever, &c. He returned home in this state, and by means of fomentations, of anodynes, of mild purges, and afterwards of baths, he entirely recovered his former health.

A young lady, in the seventh month of her pregnancy, consulted me on account of an eruption which itched a great great deal, and occasioned her sleepless nights, without any other inconvenience; I directed her to lose blood and take some cooling drinks, advised a mild and cooling regimen, and desired her to wait patiently for her accouchement, which would certainly cure her of her malady, unless it depended upon some particular cause, and I explained myself no further. The eruption resisted all the remedies I had prescribed, and the lady, impatient to be rid of it, rubbed herself, by the advice of some women, with an ointment sold in the shops as a specific for the itch. The eruption in a few days entirely disappeared, but it was followed by a hard painful tumour, the size of a walnut, within the labia pudendi, just above the meatus urinarius. I was sent for to her, and having heard this account, I did not for a moment doubt the itch having been venereal, and that her husband had communicated it to her. I mentioned this, and he confessed to me his former irregularities, and begged of me to do every thing in my power to cure his wife.

The case was too urgent to wait for the accouchement of this lady, more especially, as a few days after the appearance of the tumour, she was troubled with warts about the anus, so that she could not sit down without great pain, and she voided her urine with difficulty. I determined therefore immediately, notwithstanding her advanced pregnancy, to employ mercurial frictions; having made her lose blood, I ordered her to rub in half a drachm of ointment, containing a third part of mercury; on the following day but one I made it stronger, and thus continued increasing the dose gradually to two drachms, every other day for a month, at the end of which time she was delivered of a fine boy, perfectly healthy, and who during five years has not suffered the least inconvenience. The mother had a good getting up; the warts and tumours left her as soon as she was delivered. However, as she had used only three ounces of ointment, we thought it more safe to recur to the frictions after her confinement. She perfectly recovered her health, and from that time has felt no other inconvenience but what is common to pregnant women, having since borne several fine strong children. Her husband, after her recovery, was treated in the same manner, and perfectly cured.

The first case is a clear example of the itch arising from an internal cause, produced solely from a vitiation in the humours, brought on by the grossness and bad quality of the food and of the air; in the other, the itch also arose from a vitiation of the humours, by an extraneous poison, and was symptomatic, and from the nature of its cause may be said to be venereal.

We see in both instances how dangerous it is to attack the disease, and to repel it inwardly by topical applications, before we have removed the internal source of it, which is also confirmed by several examples related by authors.

I knew, says Zacutus Lusitanus, a man of a bad habit of body, who was subject on the approach of spring to an itch, which nature at that time threw out; he anointed himself with a composition of cerusse and litharge, and having by these means repelled the humour, or salutary evacuation, he was seized in the night with a violent dyspnœa, and died.

Amatus Lusitanus relates, that a young Florentine, seeing his body covered over with a filthy itch, would, in spite of the advice of his physicians, anoint himself with a composition containing a small quantity of arsenic, and the next day his servants found him dead in his bed.

The learned Gregoire Horstius makes mention of a young student living at a taylor's, who was attacked with an itch, and being willing to free himself from it, he anointed his skin, without having first used internal remedies; the night after he was seized with epileptic fits, on which account he was called to him with another of his colleagues; they employed the usual remedies, and he recovered.

Daniel Sennert relates the history of a student, in whom the itch was driven inwardly, who was blind for two days; afterwards he had a violent oppression and tightness in the breast, and voided urine of a very black colour, of which symptoms he was relieved by medicines which caused the itch to reappear; he particularises none of them but fumitory. The same person, three months afterwards, became ill again, without, however, losing his sight, but he was attacked with epilepsy, which was cured by the usual remedies.

The same author says also, that he knew many persons attacked with pains in the chest, with pleurisy, and many other disorders from a repelled itch, particularly a youth of 14 years, who having used some kind of ointment for the cure of an itch, passed black urine, was deprived of sight, and seized with epileptic fits, which frequently returning, at length put an end to his life.

What must be done then to free us from a disease which, besides besides being so troublesome, obliges us to avoid company, and deprive ourselves of the pleasure of society?

I answer, that in order to proceed safely and securely, we must keep in view the four distinct species of itch, which we find in practice, always observing the greatest circumspection, and must never employ external remedies too hastily to drive in the eruption, for the most fatal consequences, as we have seen, are produced by such methods.

We sometimes find in old people such a violent itching takes place, that it cannot be borne, without scratching to that degree as to rub off the skin and fetch blood; it would not be proper to give the name of *itch* to this complaint, for it is rarely accompanied by pustules; the skin is generally dry, wrinkled, rough, and covered over with small points scarcely perceptible, resembling in some measure *shagreen*. If in old age they do not experience this inconvenience, they will have some other, equally or even more troublesome; as ardor urinæ, inflamed eyes, or bleared or weeping eyes, or a frequent obstinate cough, sometimes dry, and at other times attended with expectoration of thick, yellowish, or greenish matter. These are the four usual complaints of old age; they are seldom all present at one time, but generally some one or other of them, and they all depend upon the same cause. In short, as we grow old our fibres become hard, dry and stiff, almost cartilaginous, and even nearly ossified; they are deprived by degrees of that soft, bland, lubricating fluid which renders them supple, facilitates their motion, and prevents the stiffness and rigidity of the solids, as well internally as externally.

The humours also, at the same time, become vitiated; they daily lose their bland and softening properties, they dry up, become more saline and thicker, because their motion through the vessels is slower, from the want of power in these to circulate them so freely as before. The fluids then rendered saline, and deprived of their mild qualities, slowly circulated through the solids, in some manner become stagnant, and produce an irritation which causes the itching, the *ardor urinæ*, the inflamed eyelids, or the cough and expectoration, according to the state of the part, or the effect the vitiation of the blood or lymph produces upon the fluid contained in it, or separated therefrom by it.

A young physician, when he is called to a patient who is ill from a repelled itch, must immediately determine if it is an acute or chronic disease. If acute, he must treat it according to the nature and violence of the symptoms; we must not be sparing of blood-letting to obviate internal inflammations, and must give diluents, refrigerants, mild sudorifics, and gentle purgatives and anodynes; upon these depends the cure of almost all acute disease, and the proper choice and succession of them indicate the ability of the physician.

But if the patient is labouring under a chronic disease, we must do every thing to recall the morbific matter outwardly, and this is one of the most difficult things in medicine, for the humours once displaced are not easily controuled, and can seldom be brought back to their former seat; we must however neglect no endeavours to reproduce the itch; and for this purpose we must employ mild sudorifics, in drinks, decoctions, broths, &c. If this does not succeed, we must apply blisters to the parts where it first appeared; we must also make the patient go to bed to a person affected with the itch, and wear his shirt. These means have sometimes succeeded in making the itch reappear on the skin, so that the patient has been relieved, and freed from all that he before suffered, and the itch has afterwards been cured by remedies suited to its nature and causes.

If the itch does not at all re-appear, and does not follow the sweats, we must dilute well, and drive it forth by stool and urine. Experience and daily practice show us the great consent existing between the skin, the kidneys, and the bowels; and that what cannot pass off by one way will by another. We must therefore employ purgatives and diuretics, without ever forgetting mild diaphoretics and sudorifics, which may be usefully combined together, all having a tendency to dislodge the morbific matter, and drive it outwardly.

Lastly, If the repelled itch does not give way to any of the methods we have proposed, we must open an issue upon the arm, or even a second, upon the leg, to afford a more free and uninterrupted passage to the morbific matter by these drains.

Although I have said the repelled itch produces very ill effects, I do not believe, that when it once appears on the surface of the skin, it can return inwardly; I would only be understood to mean, that the itchy matter cannot make its way through the skin when its pores are stopped by pomatum, ointments, &c. and not being suffered to pass out, is necessarily retained, and causes disease in internal parts. We may however be assured, that humours arrested by by the skin may be thrown inwardly, as we find that mercury enters the system by that mode, and even that ointments, or liniments against worms, produce their effects upon the intestines, when applied to the abdomen or navel, especially in young children.

### 3. Herpes.

This disease nearly resembles the itch, especially the first species of it, which I call the farinaceous, furfuraceous, or miliary, wherein we may perceive upon the skin something resembling meal, or very fine bran, which falling off in almost imperceptible scales, discovers the skin somewhat reddish, and covered with small points, resembling millet or mustard seed, accompanied by some heat and itching. This meal or bran, which falls off in small scales by the least rubbing, is soon after renewed, and again falls off in succession.

The second species is the crustaceous, characterized by crusts, more or less thick, which cover the affected part; they are usually dry, they separate and fall off, with some pain, leaving the skin red and tender, but new crusts soon succeed, and again fall off. This, like the former species, is accompanied by itching and heat, and even with much pungent smarting; it is dry and stationary, occupying always nearly the same place, and not spreading like the first; it does not grow larger and deeper in its progress, nor is it moist like the third species.

The third and last species is the spreading herpes, moist or running; it is red, attended with heat, inflammation, and great itching; it discharges a greyish or yellowish viscid serosity, sometimes reddish, and very hot and acrid; it corrodes all the neighbouring parts, often extending itself to a great distance. As in its progress it becomes larger and deeper, and ulcerates the part it attacks, and as it is sometimes accompanied with a sanious or purulent discharge, we give it the name of *ulcerous herpes*.

Is there no difference, it may be said, between the itch and the furfuraceous herpes, since both these diseases, according to you, have the same origin, and occupy the same seat? I answer, the only difference which appears in the symptoms or effects of these two diseases depends upon the different quality or quantity of the heterogeneous matters carried off by transpiration. If, for instance, this is very gross and dry, and charged with salts or sulphur, it will produce the dry itch and large pustules; if these principles are more diluted, it will be the moist itch; and lastly, ly, if the salts and sulphurs contained in the humours are more subtilized, and rendered volatile and active, the miliary herpes will be produced; for, indeed, upon the difference in the humours, and their principles upon the strength or weakness of our fibres, and in a word, upon their different tone and action, depend different constitutions, and thence arise the various kinds of diseases to which we are liable.

It appears from what has been said, that the herpes generally arises from an internal cause, from some depraved humours, which kind nature throws outwardly; we must therefore regard this disease as salutary; it serves as a drain for whatever is hurtful in the mass of humours, and they are thereby purified. It becomes very important therefore, not to stop this drain, but to afford an escape to the humour which appears under the form of meal or bran, for if the herpes either disappears of itself, or is repelled by topical application, there is the greatest danger of the patient falling into some violent, or even fatal disease, as will be seen in the following histories.

A man, 35 years of age, in extremely good health, had for a long time, a herpes between his thumb and fore-finger; it was furfuraceous and red underneath, not spreading, but quite at a stand; he felt no inconvenience from it but an itching, which occasioned him to rub or scratch it. Some person imprudently gave him a medicine, the composition of which I am unacquainted with; he rubbed the affected part with it, and the next day the herpes disappeared; but being called to him two days afterwards, I found him in a high fever, his face extremely red and swelled, complaining of a heaviness and pain in his head, and great heat and thirst; what is most remarkable is, there was a discharge from his face of a serosity so acrid and sharp, that it corroded whatever part it run over, as if it had been touched with liquid caustic. Seeing him in this deplorable condition, I immediately bled him, and repeated it several times, from the arm and the foot; I gave him softening emulsions and cooling laxatives and enemas; by these means, avoiding all topical application, I allayed the heat of the humours, the fever, the redness of the face, and the thirst. Afterwards he was gently purged, the fever entirely left him, the swelling of the face and discharge subsided, and warm fresh water baths, and the use of asses milk, completed the cure, preventing also the return of the herpes, which never again appeared.

A merchant, about 60 years old, very fat, and of a full gross gross habit, was subject to a furfuraceous herpes, which had continued on one of his knees many years, attended with a troublesome itching; he had often requested me to give him some medicines to relieve this, but I always refused, because I knew this herpes secured him against every other disease, and he was, except that, in perfectly good health. One day he perceived himself quite relieved from his itching, and could no longer see the herpes, which had completely disappeared, by reason of his having felt some chagrin on some occasion; but it only raged the more vehemently within, for in four days afterwards he was seized with such violent convulsions of the muscles of the thorax, and such acute pain, that he could scarcely move or breathe, and uttered doleful cries. Being called to him, I found him in a complete tetanus; I bled him both in the arm and foot, and although he had no fever, the blood always appeared very sizy. I also gave him mild sudorifics, laxative glysters, and anodynes, and without having recourse to the affusion of cold water upon the thorax, as recommended by some practitioners, I had the satisfaction of seeing the pain and oppression in the chest daily diminish, and the power of motion increase; but as the disease did not completely go off, I thought it right to recall the herpes to the knee which it had previously occupied; for this purpose I covered it with a vesicatory, and when the discharge took place, the pains and oppression which he still felt in a small degree, completely left him, the herpes re-appeared, and the patient recovered perfect health. He continued quite well for about five years, when the herpes again disappeared, whether spontaneously, or from what cause, I do not know; the patient became drowsy and fell into an apoplexy, from which he could not be recovered.

